# The Formation of Melanocyte Apoptotic Bodies in Vitiligo and the Relocation of Vitiligo Autoantigens under Oxidative Stress

**DOI:** 10.1155/2021/7617839

**Published:** 2021-10-28

**Authors:** Jun Tian, Yaojun Wang, Ming Ding, Yue Zhang, Jiaoni Chi, Tao Wang, Bin Jiao, Zhe Jian, Xiuli Yi, Ye Huang, Ling Liu, Kai Li, Jiaxi Chen, Gang Wang, Tianwen Gao, Chunying Li, Qiang Li

**Affiliations:** ^1^Department of Dermatology, Shaanxi Provincial People's Hospital, Xi'an, China 710068; ^2^Hebei North University, Zhangjiakou, China 075000; ^3^Air Force Clinical College (Air Force Medical Center) of Anhui Medical University, Beijing, China 100142; ^4^Department of Dermatology, Air Force Medical Center, PLA, Beijing, China 100142; ^5^Department of Dermatology, Armed Police Forces Beijing General Hospital, Beijing, China 100027; ^6^Department of Dermatology, Xijing Hospital, Fourth Military Medical University, Xi'an, China 710032

## Abstract

**Background:**

Oxidative stress has a vital role in the early stages of vitiligo. Autoantigens released from apoptotic melanocytes (MC) under oxidative stress are involved in the presentation and recognition of antigens. However, the transport of autoantigens to the cell surface and their release to the extracellular environment are still unclear. Apoptotic bodies (ABs) have always been considered as a key source of immunomodulators and autoantigens. Yet, the role of ABs in the immune mechanism of vitiligo is still unknown.

**Purpose:**

To explore whether MC's autoantigens translocate into ABs during oxidative stress-induced apoptosis and study the molecular mechanisms underlying autoantigen migration and AB formation.

**Methods:**

PIG3V (an immortalized human vitiligo melanocyte cell line) were treated with H_2_O_2_, and ABs were separated. Transmission electron microscopy, flow cytometry, Western blot, mass spectrometry, and other methods were used to determine the relocation of specific antigens in PIG3V cells to ABs. After pretreatment with specific inhibitors (Rho kinase (Y-27632), myosin light chain kinase (MLCK, ML-9), pan-caspase (zVAD-FMK), and JNK (SP600125)), the pathway of autoantigen translocation into ABs and the formation of apoptotic bodies were determined.

**Results:**

When treated with 0.8 mM H_2_O_2_, ABs were released from these cells. Autoantigens such as tyrosinase-related protein 1 (TYRP-1) and cleavage nuclear membrane antigen Lamin A/C (Asp230) were concentrated in ABs. The expression of autoantigens and the formation of ABs increased in a time- and dose-dependent manner after treatment with H_2_O_2_, while the application of specific inhibitors inhibited the formation of apoptotic bodies, i.e., the expression of antigens.

**Conclusion:**

Vitiligo autoantigens translocate into ABs in the process of apoptosis induced by oxidative stress. The cytoskeletal protein activation pathway and the JNK-related apoptosis pathway are involved in the transport of autoantigens and the formation of ABs. ABs may be the key bridge between MC cell apoptosis and cellular immunity.

## 1. Introduction

Oxidative stress has a critical role in the activation of autoimmunity related to vitiligo [1]. Melanocyte oxidative stress produces damage-related molecular patterns (DAMPs) or autoantigens initiating an immune response and, in turn, leads to melanocyte apoptosis through the inflammatory cascade [2]. However, so far, the interaction between these two mechanisms has not been fully understood. We speculate that reactive oxygen species (ROS) are involved in presenting self-antigens, including the release of antigens from apoptosis of MCs and phagocytosis by antigen-presenting cells (APC). Apoptotic bodies (ABs) constitute an important bridge for autoantigens' release, recognition, and phagocytosis. In systemic lupus erythematosus (SLE), ABs contain autoantigens, such as heat shock proteins (HSPs), histones, and *α*-enolase, which become immunogenic after they are swallowed by phagocytes [3–5]. Under severe oxidative stress conditions, cell components and autoantigens migrate to the cell surface to form membrane vesicles, a process also known as apoptotic hemorrhage. These vesicles are then separated from late apoptotic cells and form ABs. Studies have also indicated that ABs carrying DAMPs might have a substantial role in the induction and persistence of inflammation [6]. These studies showed that ABs potentially contribute to autoimmunity by loading autoantigens and danger signals. However, in the process of ROS-induced MC cell apoptosis, the relationship between the formation of ABs and the transport of autoantigens remains unclear in vitiligo. In this study, we analyzed the possible translocation of vitiligo MCs into ABs under the induction of oxidative stress, the formation of ABs, and the autoantigens of MCs.

## 2. Materials and Methods

### 2.1. Cell Culture

Adherent PIG3V cells (Loyola University Chicago) were routinely maintained in 254 medium (Cascade Biologics, Portland, OR) containing human MC cell growth supplement HMGS-2 (Cascade Biologics), 10% FBS (Gibco, Burlington, Canada), and 1% penicillin/streptomycin (Invitrogen) at 37°C in a humidified atmosphere of 5% CO2. Before the experiment, cells were randomly divided into the control group and the H_2_O_2_ group.

### 2.2. Induction and Detection of Apoptosis

The PIG3V cells were inoculated in a 6-well plate (3 × 10^6^ cells per well) for 24 to 48 hours. After each time point, the supernatant was discarded, and 1.5 ml 254 medium was added to each well. Cells were then incubated with 0, 0.4, 0.8, 1.6, and 3.2 mM H_2_O_2_ (Sigma, China) to induce apoptosis. After 24 hours of treatment, according to the manufacturer's protocol, the V-FITC apoptosis detection kit (BD, USA) was used to distinguish between apoptotic and necrotic cells. The apoptosis rate was further measured by flow cytometry (Beckman Coulter, USA).

### 2.3. Separation of ABs

Approx. 3 × 10^6^ PIG3V cells were plated in a 75 mm^2^ flask until reaching a logarithmic growth phase. Cells were then incubated with 0, 0.4, 0.8, 1.6, and 3.2 mM H_2_O_2_ (Sigma, China) to induce apoptosis. Twenty-four hours after the induction of cell apoptosis, 12 ml of each cell culture sample was collected in a 15 ml sterile centrifuge tube (Corning) and centrifuged at 300 g twice for 10 minutes each time to remove the cell pellet. Cells were then filtered through a 5.0 *μ*m pore size filter (Millipore Co., Cork, Ireland) under gravity. The supernatant was then centrifuged for another 20 minutes at a rotation speed of 2,000 g. The cystic pellets containing apoptotic bodies were stored in a clean 1.5 ml microcentrifuge tube at -80°C until needed.

### 2.4. Transmission Electron Microscopy

The precipitate obtained from differential centrifugation and filtration was used for transmission electron microscopy. The supernatant was removed and kept at 4°C overnight. The precipitate was fixed with PBS buffer containing 2% paraformaldehyde (Sigma-Aldrich) and 2% glutaraldehyde (Sigma-Aldrich) and placed at room temperature for 10 min. Then, the pellet was washed with PBS buffer and fixed with 1% OsO4 (Taab; Aldermaston, Berks, UK) for 1 hour. Finally, the samples were dehydrated in a series of graded ethanol and embedded in epoxy resin (Epon 812). Semithin and ultrathin sections were cut on an Ultracut E microtome (Leica, Nussloch, Germany). The semithin sections (1 mm) were stained with toluidine blue (Sigma-Aldrich) and examined under an optical microscope. Ultrathin sections (60-80 nm) were stained with 2% uranyl acetate and lead citrate, and the samples were examined under an FEI Morgagni 268(D) transmission electron microscope (FEI NanoPorts, Hillsboro, USA).

### 2.5. Protein Expression Analysis

Cells and apoptotic bodies were homogenized in a cell lysis reagent (Runde Biologics) containing a protease inhibitor cocktail (Roche), lysed on ice for 30 minutes, and then centrifuged at 16,000 g at 4°C for 5 minutes. The supernatant was then transferred to a 1.5 ml microcentrifuge tube. The total protein concentration of each sample was measured using a BCA protein assay kit (Beyotime), according to the manufacturer's instructions. For SDS-PAGE analysis, a reduced protein sample was prepared by adding NuPAGE LDS sample buffer (Invitrogen) and NuPAGE reducing agent (Invitrogen) to a 20 mg extracted protein sample after which a 4-20% SurePAGE (Genscript) was performed. The separated protein was then transferred from the SDS-PAGE gel to a polyethylene difluoride membrane (Millipore, Billerica, MA). Samples were then incubated with the following antibodies: anti-Lamin A (1 : 5000, ab8980, Abcam), anti-Lamin A/C (Asp230) (1 : 10000, ab108595, Abcam), anti-tyrosinase (1 : 5000, ab170905, Abcam), anti-TRP1 (1 : 1000, ab235447, Abcam), anti-TRP2 (1 : 1000, ab74073, Abcam), anti-MCHR-1 (1 : 1000, 10163-1-AP, ProteinTech Group), anti-histone H1 (1 : 1000, 19649-1-AP, ProteinTech Group), anti-histone H2A (1 : 1000, 10856-1-AP, ProteinTech Group), anti-La/SSB (1 : 1000, 11720-1-AP, ProteinTech Group), anti-HSP70 (1 : 5000, 10995-1-AP, ProteinTech Group), anti-HSP90 (1 : 5000, 13171-1-AP, ProteinTech Group), anti-GAPDH (1 : 5000, CW0100, CWBIO Biotechnology), or anti-beta actin (1 : 5000, 20536-1-AP, ProteinTech Group) at 4°C overnight and then with horseradish peroxidase-conjugated anti-rabbit IgG (1 : 10000, Santa Cruz Biotechnology) or anti-mouse IgG (1 : 10000, Santa Cruz Biotechnology) at room temperature for 2 hours. The bound antibody was visualized using a chemiluminescence detection kit (KPL, Gaithersburg, MD).

### 2.6. Liquid Chromatography Tandem Mass Spectrometry (LC-MS/MS) Extraction, Sample Preparation Procedure, and Data Analysis

LC-MS/MS extraction and sample preparation were described in Supplementary Method 1; the procedure was described in Supplementary Method 2, and data analysis was shown in Supplementary Method 3.

### 2.7. Flow Cytometric Analysis

PIG3V cells were seeded into a 6-well plate (3 × 10^6^ cells per well). After treatment (see above), cells were assessed using the corresponding commercial kit according to the manufacturer's protocols. Cells were sorted by a flow cytometer (BD FACSVerse, CA, USA). The morphological differences of cells and apoptotic bodies were detected by FSC/SSC flow cytometry analysis.

### 2.8. Statistical Analysis

All experiments were repeated three times, and GraphPad Prism 8.0 (GraphPad Software, San Diego, California) was used for statistical analysis. One-way analysis of variance, Dunnett's test, or two-way analysis of variance were used to analyze experiments with more than two groups. Multiple comparison tests of the least significant difference were performed to compare the differences between the two groups. All groups were tested against the control group for reference. For experiments with only two groups, a paired and unpaired *t*-test was used. *P* value of < 0.05 was considered to be statistically significant.

## 3. Results

### 3.1. Isolation and Identification of MC-Derived ABs

**Figure 1:**
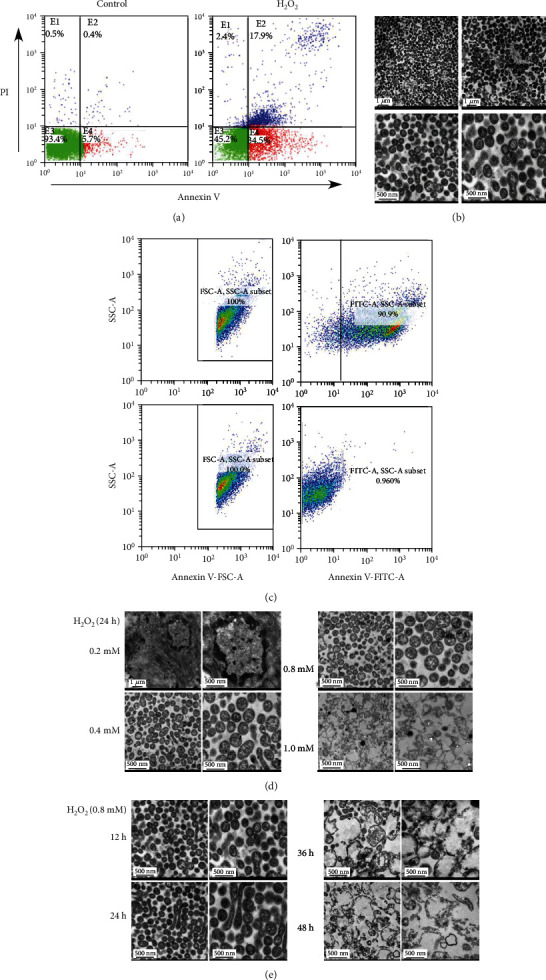
Vitiligo melanocytes induced by hydrogen peroxide (H_2_O_2_) could promote the formation of apoptotic bodies (ABs). (a) Cells were stained with annexin V and propidium iodide (PI) for 24 hours and analyzed by flow cytometry (FCM). Specific concentrations of H_2_O_2_ were chosen according to the induction of most apoptosis (annexin V+/PI- and annexin V+/PI+), before statistically significant necrosis (annexin V-/PI+; apoptosis rates: 52.4%). (b) Transmission electron microscopy (TEM) showing ABs isolated by differential centrifugation and gravity-driven filtration from the culture supernatant of PIG3V cell cultures. Apoptotic bodies are spherical, about 500 nm in diameter. (c) After induction of apoptosis and AB isolation, the percentage of particles showing a PS exposure (annexin V+) was quantified. FACS analysis was performed after the indicated incubation periods. The data shows primary data of one representative experiment. (d, e) Morphology change of ABs during different H_2_O_2_ concentrations and different time-induced apoptosis in PIG3V cells was observed by TEM. After induced with 0, 0.4, 0.8, and 1.0 mM H_2_O_2_ for 24 h, respectively, most particles were membrane-bound and spherical structures below 1.0 mM. At 1.0 mM H_2_O_2_, particles were irregular and disrupt. After induced with 0.8 mM H_2_O_2_ for 12, 24, 36, and 48 h, respectively, no structure and membrane-bound altered before 24 h. After 24 h, particles became swelling and lost membrane integrity gradually.

PIG3V cells were cultured in 0, 0.4, 0.8, 1.6, and 3.2 mM H_2_O_2_ for 24 hours to induce early cell apoptosis. When H_2_O_2_ stimulation was higher than 0.8 mM, the PIG3V cell necrosis rate was significantly increased (*P* < 0.05), and it was dose-dependent (Figure S1). Thus, we selected 800 *μ*M H_2_O_2_ concentration for further analysis (Figure 1(a)). In this study, ABs were successfully separated by multistep differential centrifugation and gravity-driven filtration from early apoptotic MCs. The morphological characteristics of apoptotic bodies were analyzed by transmission electron microscopy (TEM), and the functions of apoptotic bodies were analyzed by flow cytometry.

The morphological characteristics of apoptotic vesicle were identified by TEM. ABs are extracellular vesicles with a diameter of 100-5000 nm and are mainly characterized by vesicular structures wrapped in phospholipid bilayer [7]. In this study, the ABs were morphologically identified by TEM. The results showed that the AB samples were approximately 0.5 *μ*m in diameter, had fully structured spherical vesicles, and had a phospholipid bilayer structure, which is consistent with the characteristics of ABs (Figure 1(b)).

The surface phosphatidylserine (PS) of apoptotic vesicles was identified by flow cytometry. PS is highly expressed on the membrane surface of ABs as a key feature of ABs [8, 9], and to further characterize the quality of the collected ABs, we stained them with annexin V and analyzed them by flow cytometry [10], and the results suggested that the results showed that more than 90% of the examined particles were positive for annexin V (Figure 1(c)), suggesting that the majority of the particles we collected were from ABs.

Next, the morphological changes of ABs obtained by using different concentrations and durations (12, 24, 36, and 48 hours) were observed by TEM. When the H_2_O_2_ was <1.0 mM, the particles showed a membrane-bound spherical structure; when it was >1.0 mM, the particles showed an irregularly broken state within 24 hours. In addition, after induction with 0.8 mM H_2_O_2_ for 12, 24, 36, and 48 hours, no changes in structure and membrane binding were seen before 24 hours. After 24 hours, the particles showed gradual swelling and loss of membrane integrity (Figures 1(d) and 1(e)). These data indicated that the membrane integrity of ABs was significantly related to the level and duration of H_2_O_2_-mediated oxidative stress. Taken together, ABs may be formed during cell apoptosis under oxidative stress.

### 3.2. MC Autoantigens Were Transferred into ABs

**Figure 2:**
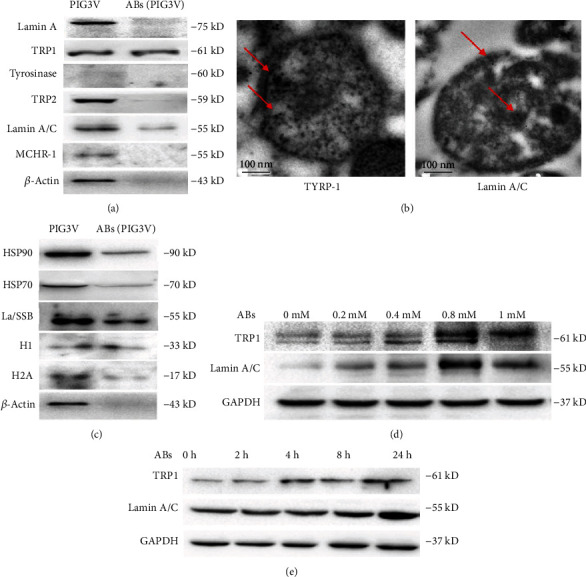
Autoantigens were translocated into apoptotic bodies (ABs). (a) ABs isolated from 0.8 mM H_2_O_2_ for 24 h treated cells were harvested and resuspended in lysis buffer. In parallel, lysates of apoptotic cells were analyzed. Then, Western blot analyses were performed as described in Materials and Methods. The detected vitiligo antigen proteins were indicated in the figure. (b) TRRP-1 and Lamin A/C (Asp230) were detected the colocalization with ABs through the immuno-electron microscope. (c) The distribution of subcellular localization of AB antigens before blebs was identified by Western blot. H1, H2A, La/SSB, HSP 70, and HSP90 were detected in ABs. (d, e) TRRP-1 and Lamin A/C (Asp230) were evaluated in lysates of ABs at 0, 0.2, 0.4, 0.8, and 1.0 mM of H_2_O_2_ treatment of PIG3V cell. Then, lysates of ABs were prepared after the indicated incubation periods, and antigens were detected by Western blot analysis. The upper Western blot shows the corresponding internal reference protein *β*-actin.

Western blot and proteomic analysis based on mass spectrometry were used to identify autoantigens. ABs were separated from the supernatant of PIG3V cells treated with H_2_O_2_. Some known autoantigen candidate antibodies, including Lamin A, TYRP, TRP-1, TRP-2, and MCHR-1, were used in Western blot analysis. During apoptosis, Lamin A is cleaved by Caspase-6 at amino acid Asp230 [11, 12]. Therefore, in this study, Lamin A/C (Asp230) antibody was also used. Our results showed that TYRP-1 and Lamin A/C were significantly increased in the AB lysate of PIG3V cells (Asp230) (Figure 2(a) and Figure S2). Immunogold electron microscopy showed the relocation of TYRP-1 and Lamin A/C with ABs (Figure 2(b)). Moreover, after treatment with different concentrations of H_2_O_2_, the protein content of vitiligo autoantigens in ABs increased significantly (Figure 2(d)). In addition, at different time points after H_2_O_2_ treatment, the protein content of TRP-1 and Lamin A/C in ABs increased significantly (Figure 2(e)).

To study the other components of ABs, in addition to vitiligo autoantigens, we used some antibodies against nuclear antigens, including histone 1 (H1), histone 2A (H2A), and ribonucleoprotein La/SSB, which were detected by Western blot. Analysis showed that the above antigens were also translocated into ABs (Figure 2(c)). In addition, similar results were observed with cytoplasmic proteins such as heat shock protein (HSP) 70 and HSP 90 (Figure 2(c)). These proteins may be involved in activating DCs; they can absorb, process, and present antigens more efficiently [13, 14]. To confirm these proteins, we used liquid chromatography tandem mass spectrometry (LC-MS/MS) to determine the components of ABs. The detected proteins were also determined by mass spectrometry analysis and by protein search and blasting database (Table 1). Collectively, these data indicated that MC-specific antigens may be present in ABs and relocate to ABs along with nuclear antigens and cytoplasmic autoantigens.

### 3.3. Cytoskeleton Activation, JNK, and Caspase Activities Participate in the Loading of Vitiligo Autoantigens into Cells in Apoptotic Bodies in a Time- and Dose-Dependent Manner

**Figure 3:**
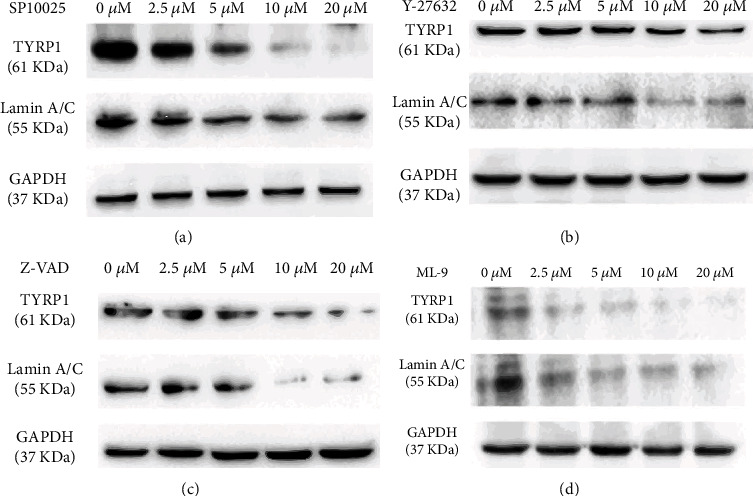
Cytoskeletal activation, JNK, and caspase were required for the loading of apoptotic bodies (ABs) with melanocyte antigens. (a–d) The sorting of melanocyte autoantigens into apoptotic bodies was affected during apoptosis in the presence of an inhibitor of JNK inhibitor (SP600125), pan-caspase inhibitor (zVAD-FMK), RHO-kinase (Y-27632), and myosin light chain kinase (MLCK) inhibitors (ML-9). The upper Western blot shows the corresponding GAPDH. One representative experiment is shown.

To compare the expression levels of ABs autoantigens, we used some specific pathway inhibitors, including Rho kinase (Y-27632), myosin light chain kinase (MLCK, ML-9), pan-caspase (zVAD-FMK), and JNK (SP600125). PIG3V cells were pretreated with different inhibitor doses and then treated with 800 *μ*M H_2_O_2_ for 24 h. Western blot showed that all inhibitors significantly downregulated the expression of autoantigens in ABs (Figures 3(a)–3(d)). These results indicated that in the process of apoptosis induced by oxidative stress, autoantigens relocated to ABs and mainly relied on actomyosin activation and JNK-induced caspase cascade.

### 3.4. The Cascade of Cytoskeleton Proteins, JNK, and Caspases Were Involved in the Formation of ABs

**Figure 4:**
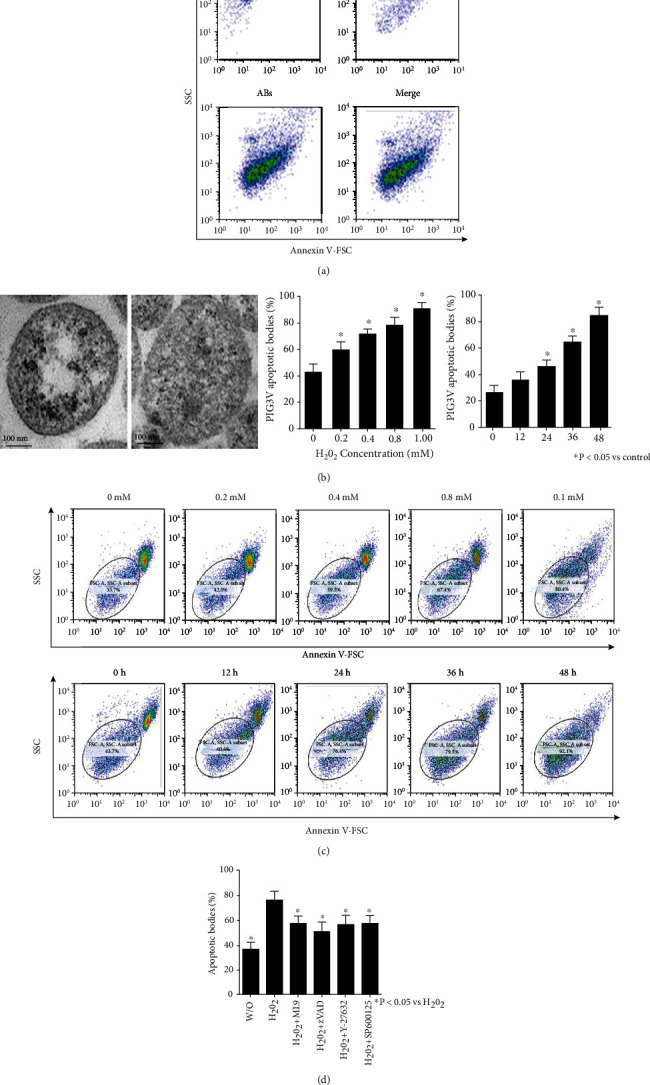
The formation of apoptotic bodies (ABs) depends on the time and concentration of H_2_O_2_ inducing apoptosis and was inhibited by cytoskeleton activation inhibitors, JNK, and caspase. (a) After induction of apoptosis, subcellular fragments were isolated by filtration and ultracentrifugation. Thereafter, isolated cells and ABs were analyzed by flow cytometry. FSC/SSC properties show standard-sized microbeads 500 nm in the upper dot plots (left) and cells (right). The lower dot plots show ABs (left) and apoptotic cells and ABs (circled population) after induction of apoptosis by H_2_O_2_ (0.8 mM, 24 h). ABs can be separated from cells by flow cytometry. Their size can be determined to about 500 nm. (b) Transmission electron microscopy (TEM) of isolated ABs reveals that isolated subcellular fragments were membrane-coated vesicles. Their size was determined to about 500 nm, consistently with flow cytometric analysis (black bar: 100 nm). (c) Formation of ABs occurs dose and time-dependently after apoptosis induction. PIG3V cells were induced to undergo apoptosis by H_2_O_2_. After the indicated incubation concentrations and periods (*x*-axis), the amount was quantified by flow cytometry. On the *y*-axis, the ratio of ABs is shown. (d) Apoptosis was induced in PIG3V cell by H_2_O_2_ (0.8 nM, 24 h) after pretreated with ML-9, zVAD-FMK, Y-27632, and SP600125. After incubation for 24 h, the amount of ABs was quantified by flow cytometry. The ratio of ABs is shown. ML-9, zVAD-FMK, Y-27632, and SP600125 significantly inhibited AB formation. Data were obtained from three independent experiments. One-way analysis of variance (ANOVA) followed by Dunn's multiple comparison test was performed, yielding a *P* value for all comparisons. ^∗^*P* < 0.05.

To analyze the formation process of ABs, we induced PIG3V cell apoptosis through H_2_O_2_. After the induction of apoptosis, the cells and ABs were harvested as described above and analyzed by flow cytometry at different stages. The subcellular fragments were separated from the cell components (Figure 4(a)). By comparison with standard-sized beads, the average size of the separated particles was about 500 nm, which was consistent with the TEM analysis result (Figure 4(b)). The formation of subcellular fragments depends on the time and dose of H_2_O_2_ (Figure 4(c)). We observed that ML-9, zVAD-FMK, Y-27632, and SP600125 could significantly inhibit the formation of apoptotic bodies, and zVAD-FMK had the highest inhibitory efficiency (Figure 4(d)). In conclusion, we confirmed that the cascade of cytoskeleton proteins, JNK, and caspases might be involved in the formation of ABs.

## 4. Discussion

Oxidative stress and immune response are two key factors in the pathogenesis of vitiligo [15]. Recent studies have confirmed that oxidative stress is the leading factor in MC apoptosis and immune damage in vitiligo [16, 17]. Oxidative stress can activate JNK and initiate the caspase kinase cascade, leading to the apoptosis of MCs [18–20], which was also demonstrated in the present study. MC-specific antigens exhibit immunogenicity and induce immune responses, leading to damage to immune MCs under certain conditions [21, 22]. However, the mechanism through which these autoantigens activate humoral and cellular immune responses remains unclear. In this study, we found that oxidative stress could induce the apoptosis of MCs, thereby promoting the shedding of ABs. We discovered that autoantigens were transferred from cells to Abs and that the signal pathways activated by JNK, caspase, and cytoskeleton proteins participated in the formation of ABs and the transport of antigens.

Apoptosis is a process of programmed cell death characterized by chromatin condensation, nuclear division, cell atrophy, and cell division into ABs, which are 500 nm in size [23, 24]. ABs represent fragments of dead cells [25], which are the molecular patterns, cytokines, autoantigens, and tissues related to damage carriers for degrading enzymes. It has been found that oxidative stress can induce apoptosis through the cytochrome C- (Cyt-C-) mediated caspase inflammation cascade activation pathway, and in turn, lead to the formation and release of ABs [26]. Previous studies have shown that under oxidative stress, activation of c-Jun N-terminal kinase (c-Jun N-terminal kinase (JNK)), mitogen-activated protein kinase (mitogen-activated protein kinase (MAPK)), and other signaling pathways start the caspase cascade and other apoptotic pathways, promote the production of ROS, and in turn, cell apoptosis [18–20]

Microvesicles' formation results from the dynamic interaction between phospholipid redistribution and the contraction of cytoskeletal proteins. It has been proven that the contractile force generated by the actin-myosin cytoskeleton structure drives membrane hemorrhage and the formation of apoptotic bodies [27–29]. RhoGTPase is a signal molecule that regulates the cytoskeleton of actin [30]. The Rho-related kinase ROCKI has been identified as the effector protein of Rho [31–33]. Activation of ROCKI, which occurs after ROCK1 binds to GTP-bound Rho [34–36], leads to an increase in the production of actin-myosin, contractility, and the formation of apoptotic bodies [37–43]. ROCKI is necessary for the redistribution of DNA fragments from the nuclear region to cell vesicles and apoptosis *in vivo*, which is consistent with our experimental results. Our experiments showed that cytoskeletal protein inhibitors could prevent the formation of apoptotic bodies, thereby inhibiting the transfer of antibodies to the apoptotic topic.

The presentation and expression of autoantigens are mediated by natural immunity, which becomes a bridge between oxidative stress and autoimmunity. ABs include core antigen components such as autoantigens, nucleosomes, and histones. The former include tyrosinase, which is the main antigen recognized by autoantibodies [44, 45]; other antigens are tyrosinase-related proteins 1 and 2 (TRP1 and TRP2), which are restricted to melanosomes [46, 47]. Lamin A is the nuclear autoantigen of systemic lupus erythematosus [48]. A/C lysis in Asp230 nuclear chromatography is another sign of apoptosis [49]. Histones are a key component of nucleosomes, and deoxyribonucleoprotein complexes are currently considered the main autoantigen targets of SLE [50]. In the early stage of apoptosis, nucleosomes migrate from the nucleus to the cytoplasm and are then exposed to the cell surface in the form of apoptotic vesicles (bleb) [51]. The formation of melanocyte self-antigens may be related to endoplasmic reticulum stress. The initiation of ER stress leads to the accumulation of immature proteins and misfolded peptides, activates the unfolded protein response (UPR), and causes the activation of the apoptotic cascade and autoimmune response [52]. Another study also confirmed that after excessive ROS, melanocytes release antigen-containing exosomes. ROS promotes autophagy of exosomes and melanosomes containing tyrosinase-melanin-A/MART-1 [21, 53]. In addition, under oxidative stress, the production and secretion of heat shock protein 70 (HSP70i) induced by melanocytes activate APC as a signal to drive the autoimmune response of vitiligo [54, 55].

In this study, we successfully separated and purified ABs from vitiligo MCs. Western blot results showed that the monoclonal antibody identified Lamin A (ASP230) and TYRP-1 in ABs. Previous studies have shown that Lamin A can be specifically cleaved into Asp230 by IL-1 converting enzyme (ICE)/caspases 6; these cleavage systems commonly occur during apoptosis [56, 57]. Our data revealed the presence of Lamin A/C (Asp230), but not Lamin A, in ABs, which indicated that Lamin A was cleaved by the caspase kinase in Asp230 during cell apoptosis and was released into extracellular ABs. These antigens, which are called ABs, are intracellular antigens of vitiligo MCs that are closely related to the severity of the disease. These results suggest that ABs are important carriers of these autoantigens.

Previous studies have shown that oxidative stress can activate JNK, thereby further activating the caspase cascade and leading to the apoptosis of MCs, which is based on the antioxidant function of mitochondria and defects in the antioxidant signaling pathway [58]. In addition, Coleman et al. [59] reported that membrane vesicles and nuclear antigens migrate to ABs during the apoptosis process of SLE and that higher levels of Rho-related protein kinase (ROCK) may have a vital role in the disease activity of SLE. Pretreatment of cells with ROCK kinase inhibitors can inhibit the formation of ABs and the migration of antigens during apoptosis [59]. In addition, the activation of the cytoskeletal protein signaling pathway can promote the migration of nuclear autoantigens to ABs during apoptosis. Accordingly, inhibiting the skeleton protein signaling pathway, and specifically inhibiting the phosphorylation of the Myosin light chain (MLC), can inhibit the migration of nuclear antigens [60]. In the process of cell apoptosis, ROCKI can be cleaved by caspase-3 in the DETD1113/G sequence to promote the formation of ABs. Accordingly, caspase inhibition or ROCK inhibits membrane blebbing and ABs formation [61]. Our results revealed that JNK, caspase, and cytoskeleton protein activation signal pathways are involved in the formation of ABs and the transport of autoantigens in the process of cell apoptosis. We speculate that factors related to oxidative stress can activate the JNK signaling pathway, further activate the caspase cascade, and then activate the ROCK protein. This further promotes the phosphorylation of MLC and participates in the formation of ABs and the migration of antigens.

**Figure 5:**
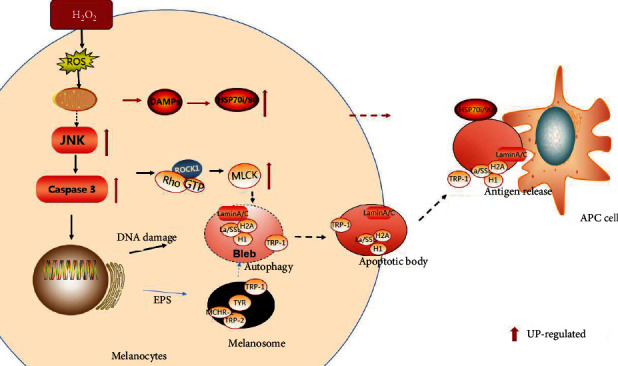
Hypothesis of the formation of melanocyte apoptotic bodies in vitiligo and the migration mechanism of vitiligo autoantigens under oxidative stress. (a) Under chronic oxidative stress, the accumulation of ROS produced by mitochondria increases, activates the JNK signal, and then activates the caspase cascade, induces apoptosis, DNA damage, and breaks, and the nucleosomes were packaged to form bleb vesicles; at the same time, the caspase cascade activates ROCKI protein, and then, MLC enzyme activation promotes the transfer of bleb vesicles to the cell membrane surface to form ABs. In addition, too much ROS induced an increase in HSP70/90. (b). Under continuous ROS stimulation, endoplasmic reticulum stress and continuous UPR activation lead to the formation of misfolded peptides involved in the synthesis of melanin; then, through the autophagy of melanosomes, a large amount of autoantigens were released; in ROCKI under the action of protein, it repositions in ABs. ABs secreted to the outside of the cell, with the participation of HSP70/90, mediate the presentation of autoantigens through APC cells.

Based on this study results and previous reports, we speculate that under oxidative stress, mitochondria generate more ROS, activate JNK signaling, and then activate the caspase cascade to induce DNA damage and breakage while nucleosomes are destroyed, after which bleb vesicles are formed. Next, the caspase cascade activates the ROCKI protein, which promotes the transfer of bleb vesicles to the cell membrane surface to form ABs. In addition, with the accumulation of ROS, melanocytes carrying DAMPs induce the production of HSP70/90, which participates in the maturation and antigen presentation of APC cells. Under continuous oxidative stress, endoplasmic reticulum stress and continuous UPR activation lead to the formation of misfolded peptides involved in melanin synthesis in melanosomes; subsequently, autophagy by melanosomes containing antigens releases a large number of autoantigens, which, under the action of ROCKI protein, are retransferred to the ABs. Finally, the ABs jointly mediate the presentation of autoantigens with the participation of HSP70/90 (Figure 5).

## 5. Conclusion

In sum, ABs in vitiligo autoantigens may have a crucial role in the immune destruction of MCs. Apoptosis and myosin light chain kinase pathways are involved in the formation of ABs and the transport of autoantigens. These findings provide a new understanding of the mechanism of vitiligo's apoptosis and immune response. Based on these findings, it is necessary to further study the autoantigens in ABs mediated by MC immune destruction to further our understanding of the relationship between oxidative stress and immune response in vitiligo.

## Figures and Tables

**Table 1:** Proteins detected in apoptotic bodies (ABs) by Western blot and identified by LC-MS/MS.

Symbol	Protein name	Number of unique peptides	Cover percent (%)	Protein molecular weight (Da)
TYRP1	Tyrosinase-related protein 1	9	27.6	60.724
LMNA	Lamin A/C	12	26.2	69.248
SSB	Sjogren syndrome antigen B (autoantigen La)	2	22.4	15.521
HSP90AB3P; HSP90AB1	Heat shock protein 90 kDa alpha (cytosolic), class B member 3	27	45.9	83.263
HSP90AA5P; HSP90AA4P; HSP90AA1; HSP90AA2; HSP90AB4P	Heat shock protein 90 kDa alpha (cytosolic), class A member 5	17	27.7	84.659
HSP90B1; HSP90B2P	Heat shock protein 90 kDa beta (Grp94)	21	33	92.468
HSPA8; HSPA2	Heat shock 70 kDa protein 8; heat shock 70 kDa protein 2	21	50	70.897
HSPA9	Heat shock 70 kDa protein 9 (mortalin)	16	33.1	72.4
HSPA5	Heat shock 70 kDa protein 5 (glucose-regulated protein, 78 kDa)	24	42.4	72.332
H2AFV; H2AFZ	H2A histone family, member V; H2A histone family, member Z	5	53.9	13.553
HIST1H1E; HIST1H1T; HIST1H1D; HIST1H1C; HIST1H1A	Histone cluster 1, H1e, H1t, H1d, H1c, H1a	3	16.9	21.364
HIST1H1B	Histone cluster 1, H1b	3	10.6	22.58

## Data Availability

No data were used to support this study.

## Additional Points

*Bulleted Statements*. What is already known about this topic? Apoptotic bodies (ABs) have been considered immune modulators and a key source of autoantigens in immunological diseases. Oxidative stress can cause melanocyte apoptosis and may be critical for the pathogenesis of vitiligo. What does this study add? ABs were released from the apoptosis of PIG3V cells (the immortalized vitiligo epidermal melanocytes) during oxidative stress, and vitiligo autoantigens including tyrosinase-related protein 1 (TYRP-1) and the cleaved nuclear membrane antigen Lamin A (Asp230) are concentrated in the ABs.

## Supplementary Materials

Supplementary Materials Figure S1: Establishment of PIG3V cell apoptosis model induced by oxidative stress and determination of optimal H_2_O_2_ concentration. (a) The cells were stained with annexin V and propidium iodide (PI) for 24 hours and analyzed by flow cytometry (FCM). (b) The bar graph represents the average value of FCM data (*n* = 3). A one-way analysis of variance (ANOVA) was performed, and then, Dunn's multiple comparison test was performed to obtain *P* values for apoptosis (annexin V + PI- and annexin V + PI +) and necrosis (annexin V-PI +). Compared with W/O, ^∗^*P* < 0.05, #*P* < 0.05. Figure S2: the relative optical density diagram corresponding to Figure 2(a). This figure shows the difference in autoantigens between PIF3V cell lysate and AB lysate. ns: the difference is not significant, *P* > 0.05.
