# Supplementary material for: The Formation of Melanocyte Apoptotic Bodies in Vitiligo and the Relocation of Vitiligo Autoantigens under Oxidative Stress
Source: Oxid Med Cell Longev. 2021 Oct 28;2021:7617839. doi: 10.1155/2021/7617839 (PMC8568525; doi:10.1155/2021/7617839)
Supplement: Supplementary Materials — Figure S1: Establishment of PIG3V cell apoptosis model induced by oxidative stress and determination of optimal H2O2 concentration. (a) The cells were stained with annexin V and propidium iodide (PI) for 24 hours and analyzed by flow cytometry (FCM). (b) The bar graph represents the average value of FCM data (n = 3). A one-way analysis of variance (ANOVA) was performed, and then, Dunn's multiple comparison test was performed to obtain P values for apoptosis (annexin V + PI- and annexin V + PI +) and necrosis (annexin V-PI +). Compared with W/O, ∗P < 0.05, #P < 0.05. Figure S2: the relative optical density diagram corresponding to Figure 2(a). This figure shows the difference in autoantigens between PIF3V cell lysate and AB lysate. ns: the difference is not significant, P > 0.05. [file 7617839.f1.zip › Supplementary Method .docx]

**Supplementary Method 1**

**Extraction and sample preparations for liquid chromatography-tandem mass spectrometry (LC-MS/MS)**

Each protein samples were processed through FASP (filter-aided sample preparation) involving buffer exchange to 8 M urea and alkylation with 50 Mm iodoacetamide prior to trypsin digestion (1:50, Promega) for overnight at 37 °C.

**Supplementary Method 2**

**Procedure of liquid chromatography-tandem mass spectrometry (LC-MS/MS)**

LC–MS/MS analyses were performed on a liquid chromatographic tandem mass spectrometry system (Ultimate 3000, Dionex, Thermo Fisher, Hemel Hempstead, UK) coupled to a linear ion trap Orbitrap hybrid mass spectrometer (LTQ OrbitrapVelos Pro, Thermo Scientific) via a nano electrospray ion source (Thermo Scientific). Technical replicates of each digested sample was injected, corresponding to a total sample amount of 3ug tryptically digested proteins. After injection, peptides were pre-concentrated with 0.1% TFA on a trap column (Acclaim® PepMap 100, 300μm × 1mm, 5μm, 100 Å). Subsequently, the analyte was transferred to the analytical column (Acclaim® PepMap RSLC, 75μm × 15 cm, C18, 2μm, 100 Å) and by using a 190 min gradient from 5 to 45% solvent B at a flow rate of 200 nl/min (solvent A: 0.1% formic acid, solvent B: 0.1% FA acetonitrile). Full-scan MS survey spectra (m/z 350−1800) in profile mode were acquired in the Orbitrap with a resolution of 120000. The 15 most intense peptide ions from the preview scan in the Orbitrap were fragmented by collision induced dissociation (normalized collision energy, 35%) in the LTQ. Data were acquired using the Xcalibur software.

**Supplementary Method 3**

**Data analysis of liquid chromatography-tandem mass spectrometry(LC-MS/MS)**

The acquired raw data were processed with the MaxQuant software (version 1.5.0.12) according to the standard workflow with additional options that match between the runs(match time window 0.5min; alignment time window 20 min),label free quantitation and intensity-based absolute quantification were selected. Database search was performed in MaxQuant with the help of Andromeda search engine against International Protein Index Human version 3.87 database (91,491 entries) with the criteria of at least one being unique, and proteins identified from the decoy database and the known contaminants were removed. Carbamidomethylation was set as fixed modification. Variable modifications included were oxidation (M), N-acetyl (protein) and pyro-Glu/Gln (N-term). The initial mass tolerance for full scans was 7 ppm and 20 ppm for MS/MS. Two missed cleavages were allowed and the minimal length required for a peptide was seven amino acids. The peptide and protein false discovery rates (FDR) were set to 0.01.
