# Supplementary figures and images for: The Formation of Melanocyte Apoptotic Bodies in Vitiligo and the Relocation of Vitiligo Autoantigens under Oxidative Stress
Source: Oxid Med Cell Longev. 2021 Oct 28;2021:7617839. doi: 10.1155/2021/7617839 (PMC8568525; doi:10.1155/2021/7617839)

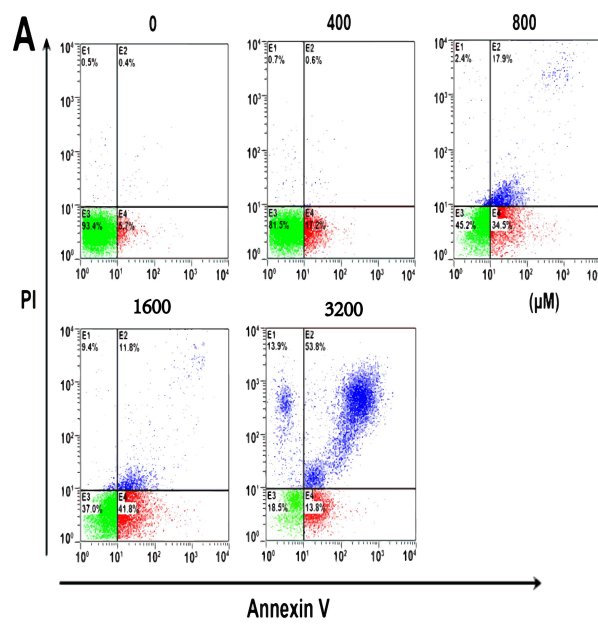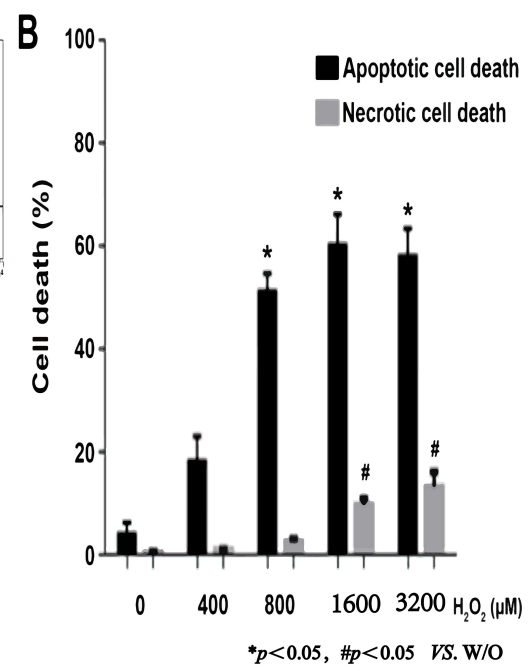

Supplement: Supplementary Materials — Figure S1: Establishment of PIG3V cell apoptosis model induced by oxidative stress and determination of optimal H2O2 concentration. (a) The cells were stained with annexin V and propidium iodide (PI) for 24 hours and analyzed by flow cytometry (FCM). (b) The bar graph represents the average value of FCM data (n = 3). A one-way analysis of variance (ANOVA) was performed, and then, Dunn's multiple comparison test was performed to obtain P values for apoptosis (annexin V + PI- and annexin V + PI +) and necrosis (annexin V-PI +). Compared with W/O, ∗P < 0.05, #P < 0.05. Figure S2: the relative optical density diagram corresponding to Figure 2(a). This figure shows the difference in autoantigens between PIF3V cell lysate and AB lysate. ns: the difference is not significant, P > 0.05. [file 7617839.f1.zip › figure S1.pdf]

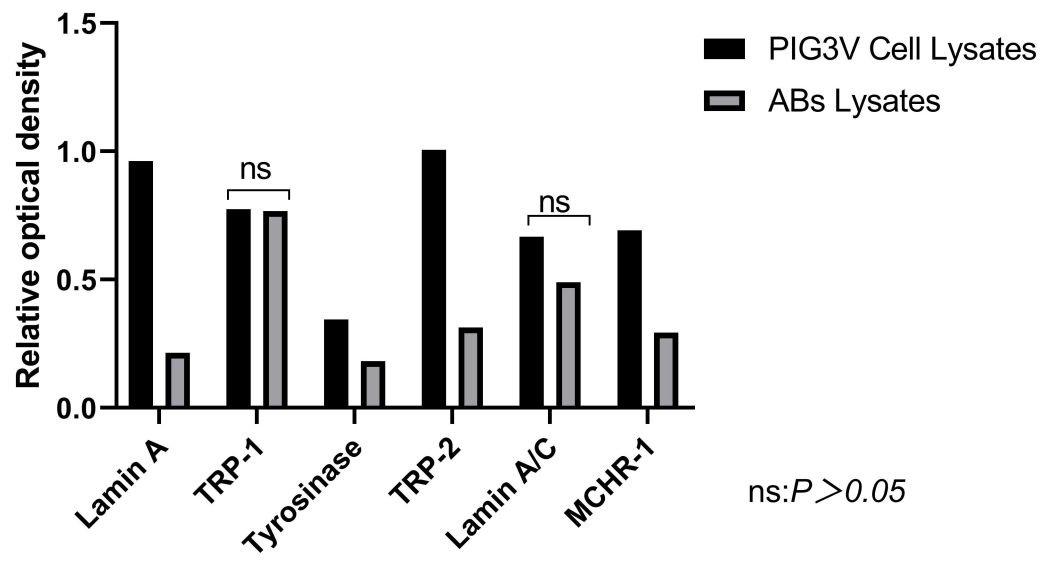

Supplement: Supplementary Materials — Figure S1: Establishment of PIG3V cell apoptosis model induced by oxidative stress and determination of optimal H2O2 concentration. (a) The cells were stained with annexin V and propidium iodide (PI) for 24 hours and analyzed by flow cytometry (FCM). (b) The bar graph represents the average value of FCM data (n = 3). A one-way analysis of variance (ANOVA) was performed, and then, Dunn's multiple comparison test was performed to obtain P values for apoptosis (annexin V + PI- and annexin V + PI +) and necrosis (annexin V-PI +). Compared with W/O, ∗P < 0.05, #P < 0.05. Figure S2: the relative optical density diagram corresponding to Figure 2(a). This figure shows the difference in autoantigens between PIF3V cell lysate and AB lysate. ns: the difference is not significant, P > 0.05. [file 7617839.f1.zip › figure S2.pdf]
